# Anatomy and Histochemistry of Seed Coat Development of Wild (*Pisum sativum* subsp. *elatius* (M. Bieb.) Asch. et Graebn. and Domesticated Pea (*Pisum sativum* subsp. *sativum* L.)

**DOI:** 10.3390/ijms22094602

**Published:** 2021-04-27

**Authors:** Lenka Zablatzká, Jana Balarynová, Barbora Klčová, Pavel Kopecký, Petr Smýkal

**Affiliations:** 1 Department of Botany, Palacký University, Šlechtitelů 27, 783 71 Olomouc, Czech Republic; zablatzka.lenka@seznam.cz (L.Z.); jana.balarynova@upol.cz (J.B.); barbora.klcova02@upol.cz (B.K.); kopecky@genobanka.cz (P.K.); 2 Genetic Resources for Vegetables and Specialty Crops, Crop Research Institute, Šlechtitelů 29, 783 71 Olomouc, Czech Republic

**Keywords:** dormancy, domestication, macrosclereids, legumes, pea, permeability, seed coat, testa

## Abstract

In angiosperms, the mature seed consists of embryo, endosperm, and a maternal plant-derived seed coat (SC). The SC plays a role in seed filling, protects the embryo, mediates dormancy and germination, and facilitates the dispersal of seeds. SC properties have been modified during the domestication process, resulting in the removal of dormancy, mediated by SC impermeability. This study compares the SC anatomy and histochemistry of two wild (JI64 and JI1794) and two domesticated (cv. Cameor and JI92) pea genotypes. Histochemical staining of five developmental stages: 13, 21, 27, 30 days after anthesis (DAA), and mature dry seeds revealed clear differences between both pea types. SC thickness is established early in the development (13 DAA) and is primarily governed by macrosclereid cells. Polyanionic staining by Ruthenium Red indicated non homogeneity of the SC, with a strong signal in the hilum, the micropyle, and the upper parts of the macrosclereids. High peroxidase activity was detected in both wild and cultivated genotypes and increased over the development peaking prior to desiccation. The detailed knowledge of SC anatomy is important for any molecular or biochemical studies, including gene expression and proteomic analysis, especially when comparing different genotypes and treatments. Analysis is useful for other crop-to-wild-progenitor comparisons of economically important legume crops.

## 1. Introduction

Seeds have two principal functions: reproduction and dispersal. There is amazing diversity in seed sizes and shapes, largely as a result of different dispersal strategies. Although the morphology of seeds varies among different plant families, the fundamental components of the embryo (cotyledons, hypocotyl, and radicle) are highly conserved. In addition to the crucial biological role of seeds in dispersal, there are also indispensable roles for animals and humans, providing most of the feed and food. In angiosperms, the mature seed consists of an embryo, an endosperm, and a maternal plant-derived seed coat (SC). Seed development involves the coordinated activities of these three genetically distinct entities [1]. The embryo represents the next generation, the endosperm nourishes and stores tissue, and the maternal plant contributes to the protective and dispersal functions of SC, and in some cases with pericarp. The maternally derived SC is responsible, in part, for the evolutionary success of the seed [2,3].

Although the SC does not take part in fertilization, its formation is dependent on the fertilization, when the SC is formed from outer (OI) and inner (II) integuments [4,5]. Cells from both integuments undergo rapid growth and differentiation upon the ovule fertilization [6] and these processes are coordinated with the endosperm [7]. The regulatory crosstalk between seed compartments has been studied in *Arabidopsis* [8,9,10]. Particularly, transparent testa mutants have indicated the control of seed dormancy and germination by the SC [8]. Furthermore, the SC plays an integral role in seed filling [11], protects the embryo, limits desiccation during dormancy and germination, and facilitates the dispersal of seeds [6,12,13]. The variability in SC anatomy is considerable and has been used taxonomically [14,15].

Legume seed development has been studied in *Medicago truncatula* Gaertn. [16,17,18,19,20,21,22,23] and cultivated pea [24,25,26,27,28,29,30,31,32,33,34]. Early in development, legume SC is a transient storage organ, which accumulates starch and proteins before storage activity starts in the embryo. When this SC storage activity is altered, embryo growth is impeded [12,35]. During seed filling, the SC is metabolically highly active and supports storage compound synthesis in the filial tissues by transmitting organic nutrients from the phloem [35]. During seed expansion and desiccation, the SC cells undergo senescence. The mature SC acts as a protective barrier for the enclosed embryo. It not only gives mechanical protection from the environment, but also mediates information from the surrounding environment, especially in relation to dormancy release and subsequent seed germination [15]. The structure and composition of the SC are essentials in these processes [13,36]. There is mechanical reinforcement through the synthesis of secondary cell walls impregnated with fats, waxes, and polyphenolic substances serving also as protectants from insect predation [30,31,37]. Recently, it was shown that the SC contains active enzymes that can persist for decades and be released upon hydration [38,39].

The domestication process has altered numerous traits [40]. In legumes, this resulted in the removal of seed dormancy, mediated by SC impermeability [40]. In our previous studies [37,41], we analyzed the anatomy of mature pea seeds in relation to dormancy release treatments (temperature and humidity oscillations). These analyses showed that the SC of non-dormant seeds of cultivated pea genotypes tend to be thinner, softer, and more elastic than those of dormant wild peas [37,41]. Similarly, there were differences in the histochemical detection of lipids and phenolic substances [37]. The current study compares the SC anatomy and histochemistry of wild and domesticated pea seeds throughout development and in relation to SC-imposed dormancy.

## 2. Results

### 2.1. Structure of Wild and Cultivated Pea Seed Coat Differ during Development

**Figure 1:**
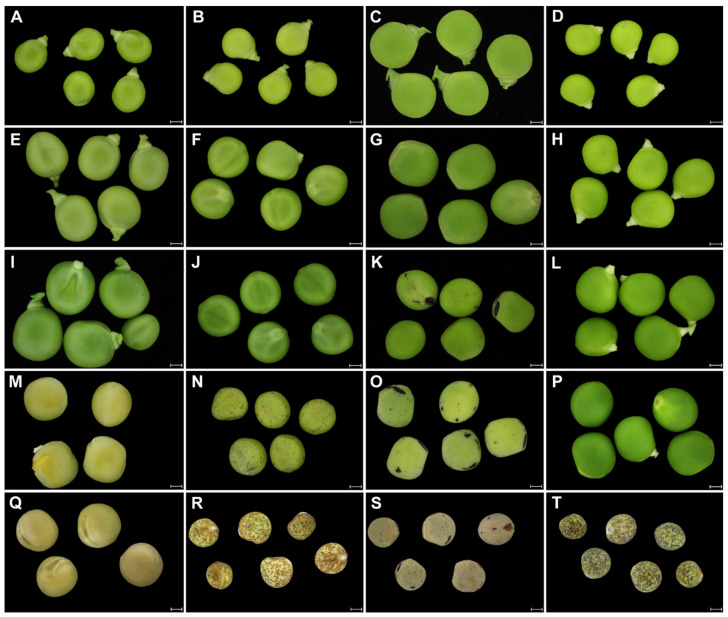
Macrographs of pea seed development. Pod dissected seeds of cultivated pea (*Pisum sativum* subsp. *sativum*): cv. Cameor (**A**,**E**,**I**,**M**,**Q**), JI92 (**B**,**F**,**J**,**N**,**R**); wild pea (*Pisum sativum* subsp. *elatius*): JI64 (**C**,**G**,**K**,**O**,**S**), JI1794 (**D**,**H**,**L**,**P**,**T**) prior to the seed coat extraction. The sampling time points: 13 DAA (**A**–**D**), 21 DAA (**E**–**H**), 27 DAA (**I**–**L**), 30DAA (**M**–**P**), and dry seeds (**Q**–**T**). Scale bars = 2 mm.

Since the aim of the study was to compare the structure and properties of the pea SC in relation to seed dormancy, we analyzed selected representative genotypes of wild and cultivated pea known to differ at the mature dry seed stage [37,41]. We were interested in when these differences are manifested during the seed development. To do this, we analyzed developmental series taken at specific time points of 13, 21, 27, and 30 DAA, and the mature dry stage (Figure 1). As shown in Figure 1, domesticated pea can either have pigmented (JI92, Figure 1N,R) or colorless SC (cv. Cameor, Figure 1M,Q). The SC pigmentation develops during seed desiccation, typically after 25 DAA, depending on the genotype (Figure 1N–P), following chlorophyll degradation. The size of the seeds increased to about 21–25 DAA, after which desiccation occurs. Interestingly, until this time-point, the funiculus was still firmly attached (Figure 1I–L), as there is ongoing nutrient transport from maternal plant. Pigmentation started to develop first at the hilum and was present as dots or patches on the SC (Figure 1K,N,O).

**Figure 2:**
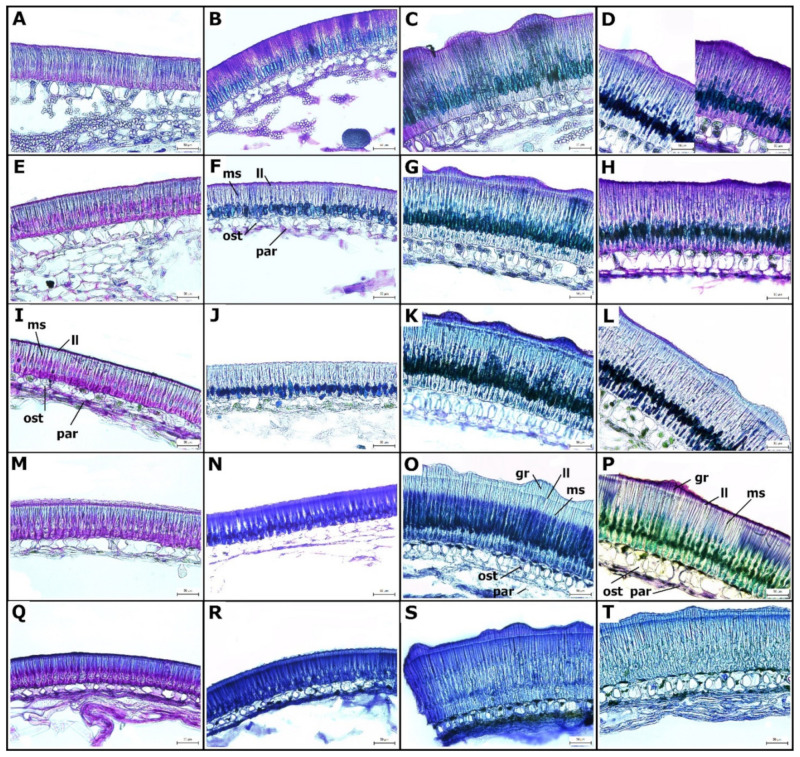
Pea seed coat structure during the development. Cultivated pea (*Pisum sativum* subsp. *sativum*): cv. Cameor (**A**,**E**,**I**,**M**,**Q**), JI92 (**B**,**F**,**J**,**N**,**R**); wild pea (*Pisum sativum* subsp. *elatius*): JI64 (**C**,**G**,**K**,**O**,**S**), JI1794 (**D**,**H**,**L**,**P**,**T**) stained with Toluidine Blue. Development series were analyzed at 13 DAA (**A**–**D**), 21 DAA (**E**–**H**), 27 DAA (**I**–**L**), 30 DAA (**M**–**P**), and dry seeds (**Q**–**T**). Staining variability within one seed segment in genotype JI1794, 13 DAA (**D**). Scale bars = 50 μm. Abbreviations: ms—macrosclereid cells, ost—osteosclereids, par—parenchyma, gr—gritty, ll—light line.

**Figure 3:**
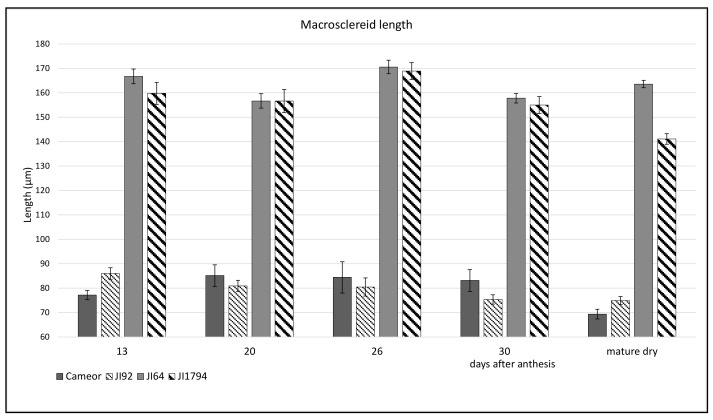
Length of macrosclereid cells during the seed coat development. Five seeds of each stage were sectioned and five sections per seed were measured. Mean and SD are shown.

Toluidine Blue (TB)-stained sections of the SC showed the differentiation of macrosclereid, osteosclereid, and parenchyma cells layers at 13 DAA. Strong metachromatic staining showed cells with a dense cytoplasm and small vacuoles, indicative of high metabolic activity. At this stage, macrosclereid cells elongated and were oriented perpendicularly to the surface. The SC thickness and structure was formed already at the 13 DAA stage when it reached its final size (Figure 2). The differences between wild and cultivated genotypes were very obvious. The wild pea SC was about twice as thick as the SC of cultivated genotypes (Figure 3) and had a rough surface with a manifested gritty trait [42], while the SC of the cultivated pea was thin (thickness of 80–90 µm) and smooth (Figure 2 and Figure 3). This difference is mainly due to macrosclereid’s length, while osteosclereids contributed only little (Figure 3).

The terminal part of the macrosclereid cells is optically separated from the rest of the cell by the so-called “light line” (LL). The light line was already apparent at 13 DAA and became more significant with development. It was present both in cultivated as well as wild pea genotypes, but was more pronounced in wild pea genotypes (Figure 2). Thus, the macrosclereid content had a bottle-like shape (Figure 2) with remarkably thick walls at maturity.

Also at 13 DAA, stage cells of the sub-epidermal layer, located below the epidermis, were already differentiated into osteosclereid cells, with the secondary wall developed and forming constricted curvatures in the central part of the cell. Osteosclereids were fully developed at the 21 DAA stage. This was marked by an increase in cell wall thickness, especially in wild pea genotypes (Figure 2C,D,G,H). This osteosclereid layer had extensive intercellular spaces (Figure 2). Both macrosclereid and osteosclereid layers contribute to the mechanical strength of the SC.

The underlying layer consists of loosely arranged parenchyma cells, characterized by intercellular spaces. These are nutritional tissues, which gradually collapsed from 13 DAA during the seed maturation. At younger (13–21 DAA) stages some of these cells (chlorenchyma) contained chloroplasts and starch granules (Figure 2I–L). With the expansion of cotyledons of the embryo, a large part of parenchyma is crushed and compressed, forming a boundary between the living cells of the SC (osteosclereids and macrosclereids) and the cotyledons of the embryo. The most proximal parenchyma cells were loosely attached by 27 DAA and consequently are typically lost during sample preparation, especially cry-sectioning. These branched parenchyma cells form nutritional tissue, which gradually collapses during seed maturation as a consequence of pressure exerted by the expanding cotyledons. In peas, there is no endosperm or aleurone cell layer.

### 2.2. Cell Wall Lipids Staining with Sudan Red

**Figure 4:**
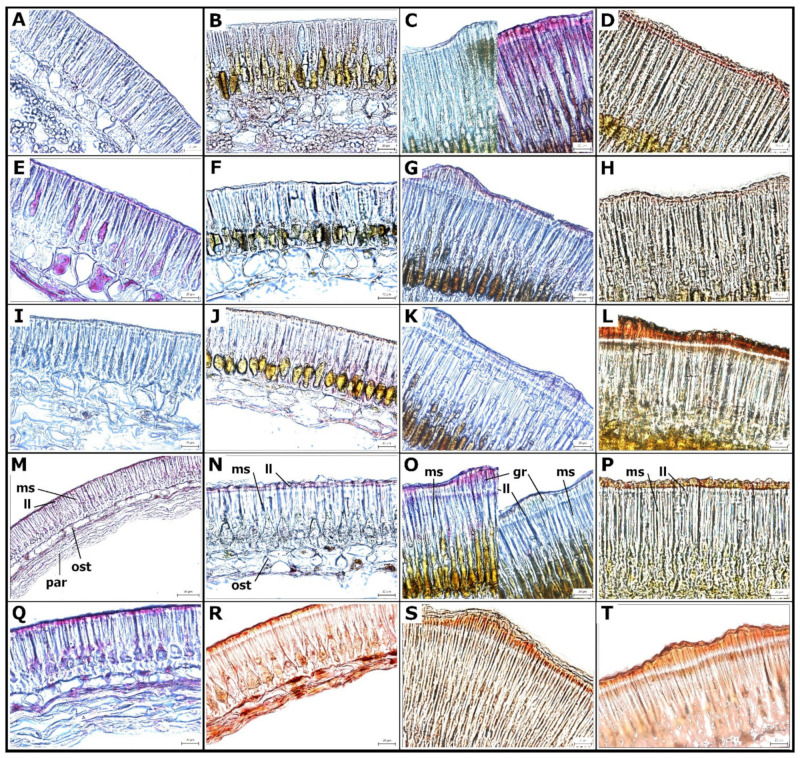
Histochemical Sudan Red staining of lipidic substances. Cultivated pea (*Pisum sativum* subsp. *sativum)*: cv. Cameor (**A**,**E**,**I**,**M**,**Q**), JI92 (**B**,**F**,**J**,**N**,**R**); wild pea (*Pisum sativum* subsp. *elatius*): JI64 (**C**,**G**,**K**,**O**,**S**), JI1794 (**D**,**H**,**L**,**P**,**T**). Development series were analyzed at 13 DAA (**A**–**D**), 21 DAA (**E**–**H**), 27 DAA (**I**–**L**), 30 DAA (**M**–**P**), and dry seeds (**Q**–**T**). Staining variability within one seed segment in genotype JI64, 13 DAA (**C**) and 30 DAA (**O**). Scale bars = 50 μm. Abbreviations: ms—macrosclereid cells, ost—osteosclereids, par—parenchyma, gr—gritty, ll—light line.

The surface of the SC is covered by a thin cuticle, which has a lipidic character and can be stained by Sudan Red (SR) (Figure 4), used for the detection of triglycerides, lipids, and lipoproteins. We detected no staining in any of the developmental stages of the domesticated Cameor genotype (Figure 4A,E,I,M,Q), while domesticated and non-dormant JI92 (with pigmented SC) showed staining in the basal part of macrosclereid cells from 13 DAA stage (Figure 4B,F,J,N,R). Both wild type, dormant JI64 and JI1974 genotypes had strong staining (Figure 4).

**Figure 5:**
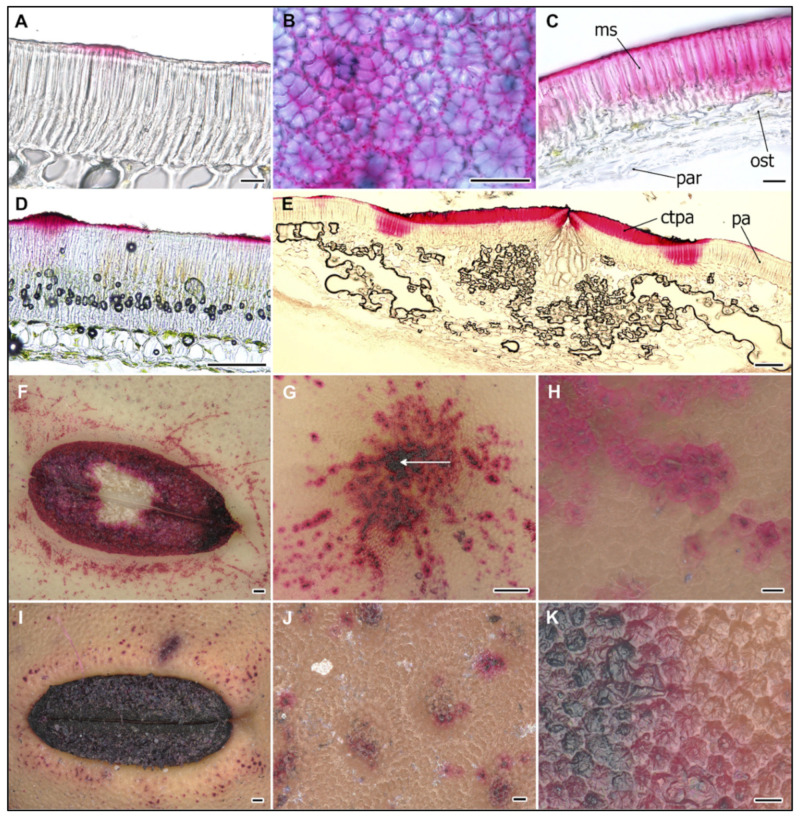
Histochemical Ruthenium Red staining of pectin substances. Mature dry seed staining of cultivated pea (*Pisum sativum* subsp. *sativum*): cv. Cameor (**A**–**C**), paradermal section (**B**), 24 h imbibed seed (**C**); wild pea (*Pisum sativum* subsp. *elatius*): JI64 (**D**,**E**), hilum region (**E**). Surface of cv. Cameor (**F**–**H**), hilum (**F**), micropyle, arrow (**G**); JI64 (**I**–**K**). Scale bars = 20 μm (**A**–**C**), 100 μm (**D**–**G**,**I**) and 10 μm (**H**,**J**,**K**). Abbreviations: ms—macrosclereid cells, ost—osteosclereids, par—parenchyma, ctpa—counter-palisade, pa—palisade cells.

In addition, from 27 DAA there was a clear signal above the light line, particularly in the JI1794 genotype. At the hilum region, SR staining of counter-palisade cells started from 27 DAA in JI64 and JI1794 wild pea and was found in the JI92 cultivated pea but only at the very surface. Strong SR staining of the parenchyma and asterosclereids was detected in all development stages of all genotypes except for Cameor. In the hilum region, SR staining was strong in the lower part of parenchymatic cells, particularly in the region close to vascular bands and around the micropyle throughout cultivated pea SC development (not shown). Notably, all staining methods detected non-homogeneity of the SC, both in wild and cultivated genotypes (Figure 5). This might relate partly to the SC pigmentation pattern, except of non-pigmented cv. Cameor.

### 2.3. Ruthenium Red Staining of Polyanionic Substances

Ruthenium Red (RR) binds to a variety of polyanions and is typical rather than specific for pectins (Soukup 2019), but is frequently used in seed biology to detect pectineaous mucilage [43]. Fixed seeds of the early developmental stages were not assessed by RR staining, as the properties of the SC are altered.

**Figure 6:**
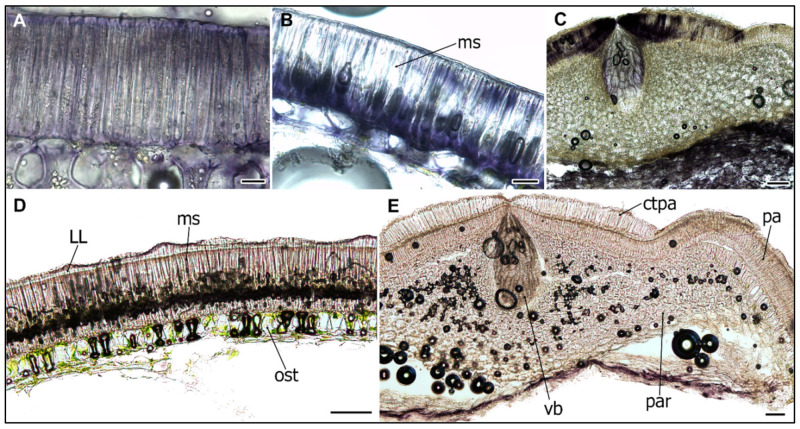
Histochemical localization of peroxidase activity in the pea seed coat. Cultivated pea (*Pisum sativum* subsp. *sativum*): cv. Cameor sampled at 13 DAA (**A**), and mature dry stage (**B**), hilum region at 25 DAA (**C**); wild pea (*Pisum sativum* subsp. *elatius*): JI1794 at 25 DAA (**D**) and hilum region of mature dry seed (**E**). Scale bars = 20 μm (**A**,**B**) and 100 μm (**C**–**E**). Abbreviations: ms—macrosclereid cells, ost—osteosclereids, par—parenchyma, ll—light line, vb—vascular bundle.

Thus, only the mature dry stages could be analyzed comparatively. RR staining was not detectable across the entire SC surface but it appeared in patches, both in cultivated (Figure 5A,F–H) and wild (Figure 5D,I–K) genotypes. It was localized to the upper part of macrosclereid cells, particularly above the LL, and to the lamella between macrosclereid cells, and to a lesser extent to the lumen. Upon seed imbibition, RR staining was detectable across the SC in cultivated genotypes (Figure 5C), while in wild genotypes only in macrosclereid cups (Figure 5D). There are several specialized structures clearly visible on the SC: the hilum, the micropyle, and the lens (or strophiole). The micropyle is a narrow gap in the integuments through which the pollen tube usually passes during fertilization. In pea seeds, the micropyle is observed next to the hilum. Interestingly, there is no macrosclereid layer in the micropyle region, only parenchyma cells (Figure 5E and Figure 6C,E).

The hilum is a specialized structure established at the place of the attached funiculus, connecting seeds to the maternal plant. In peas as in other legumes, the funiculus is fused to the outer integument and forms raphe. The hilum is also formed early, as at 10–13 DAA when the funiculus is still firmly attached, there is a clearly observable hilar groove with hilum aperture. These structures do not have significant differences between wild and cultivated pea genotypes (Figure 5F,I). In the hilum region, in addition to palisade, there is also a counter-palisade layer, a clearly developed tracheid bar, and asterosclereids. This layer has a different chemical composition and is particularly rich in hydrophilic compounds such as pectins, as detected by Ruthenium Red (Figure 5E). The macrosclereids at the hilum region form a double layer with osteosclereids modified into pockets of reticulate and thick cell walled sclerenchyma (Figure 5E and Figure 6C,E). The vasculature in the pea SC is represented only by a single chalazal vein with two lateral branches. There is a tracheid bar underneath the hilum groove, but no osteosclereid layer. The lens (strophiole) is located opposite the micropyle, rather near the hilum. In this region, the osteosclereid layer is absent. The macrosclereid cells are less variable in size and shape across the SC, except the hilum and strophiole regions, where they are typically 2–3× longer.

Micropyle and hilum regions stained strongly with RR both in wild and cultivated types. Interestingly, only counter-palisade cells stained (Figure 5E). Both cultivated genotypes (cv. Cameor and JI92) contained a high level of polyanionic cell wall components in the middle lamella of their macrosclereids (Figure 2), as revealed by metachromatic TB (Figure 2) and RR staining (Figure 5A–C). Micropyle region stained intensively with RR and SR (Figure 4 and Figure 5G), indicating the presence of pectin and lipidic substances, respectively.

### 2.4. Peroxidase Activity during the SC Development

Since peroxidases (PRX) along with laccases and polyphenol/catechol oxidases have been implicated in lignin biosynthesis and their activity was detected in the SC of soybean, we studied their localization in pea SC. PRX activity was detected in macrosclereid cells already at 13 DAA stage and increased during development until 25 DAA (Figure 6A,B,D). High PRX activity was found in the lower part of the parenchymatic cells, the counter-palisade cells of the hilum, the vascular bundle, and the micropyle regions (Figure 6C,E). In wild (JI64, JI1794) genotypes, there was PRX staining localized below the LL region (Figure 6D), while in cultivated (Cameor, JI92) genotypes, staining was more dispersed.

## 3. Discussion

Legume SC structure has been extensively studied [24,25,26,27,28,44,45,46,47]. However, these studies analyzed either mature dry seeds and/or only cultivated genotypes [34,48]. The novelty and differences occur when compared to wild pea genotypes. To our knowledge, this study is the first one conducted comparatively on wild and cultivated pea (or any wild and cultivated legume seeds) throughout the SC development. Previously, we have analyzed either the mature dry SC of wild and cultivated peas [41] or during the treatments releasing the seed dormancy [37]. In the latter study, SC development was partly analyzed but not in a systematic manner.

During development, the SC has an important function to deliver the nutrients to the developing embryo [34]. The nutrients transported by phloem are moved into SC parenchyma cells, and then released into apoplasts, followed by uptake by developing cotyledons [23]. We focused on the later stages of SC development, starting from 13 DAA. At this stage, the liquid endosperm is being consumed by the expanding embryo. Correspondingly, by 17 DAA, the majority of the seed tissue is actual embryo with growing cotyledons and embryo axis [48]. At these stages, the SC is already fully developed, both in wild and cultivated pea genotypes (Figure 2). Notably, there is large developmental progress between 13 and 17 DAAs, which agrees with the findings of van Dongen et al. [34], irrespective of different cultivars and cultivation conditions. At that time, protodermal cells are differentiated into epidermal macrosclereids. Our results obtained on cultivated pea cv. Cameor representing the modern dry field pea with a non-pigmented, smooth, and thin SC, correspond well with previous studies made on similar material [34,48]. Extensive wall thickening, vesicle trafficking, and vacuolization throughout cultivated pea SC development was observed by Harris [32,33]. Interference microscope studies indicated a sharp difference in refractive index at the LL zone. Furthermore, Harris [33] found that osteosclereids still develop after the macrosclereids are established. The lytic process creates spaces between them and elongation leads to the narrowing of the central part resulting in typical shapes of hourglass (bones, osteo-). Most commonly, studies have pointed out that the macrosclereid cell layer controls the water entry [13] and *M. truncatula* mutants with a defective outer cell layer differentiation, and in particular cuticle deposition, have supported this [49]. Cells of the macroslereid layer are rich in secondary metabolites and correlations between pigmentation and SC permeability were shown [13,19,41]. Analysis of the chemical composition of pea SC showed that polysaccharides, such as hemicellulose, cellulose, and especially pectins, comprise the largest proportion and their deposition is developmentally regulated [50]. Staining of these substances with RR did not show differences between wild and cultivated pea SC (Figure 5). The presence of lipid substances in wild types corresponds with the findings of Janská et al. [37] and confirm the results of comparative chemical analysis with detected hydroxylated fatty acids in dormant, wild types [41,51]. Within macrosclereids, there is a LL layer which has been variously interpreted and its nature still remains unresolved [24,28,52,53,54]. A more plausible interpretation is that LL is the result of the altered orientation of cellulose microfibrils or structural discontinuity between the lignified and non-lignified parts of the cell wall [55,56]. We did not observe expansion of the parenchyma cells as found by Nadeau et al. [48] at 16 DAA, compared to the 14 DAA stage. In our study, this layer gradually degenerated through the development. The discrepancy is likely due to the consistency in SC sampling and sectioning orientation. It is unlikely to have such expansion and constriction in the course of 2 days as shown by Nadeau et al. [48] and our results are consistent with the findings of van Dongen et al. [34]. We observed that the SC is not homogenous through the seeds, not only structurally with more expanded parenchyma cells in proximity to the hilum region, but also compositionally as visualized by histochemical staining (Figure 5A,D, F–K). Contrary to the study by van Dongen et al. [34], we did not observe well-developed parenchyma layers, especially in the later stages of 21 to 30 DAA. This is unlikely to be due to the cultivar (cv. Marzia vs. cv. Cameor used in our study), but due to different fixation and sectioning (acrylic polymer vs. cry-sectioning in our study) protocol.

Parenchyma cells are metabolically highly active, especially those in the proximity to the vascular bundle. These are larger than their neighbors and with intercellular spaces, which support the diffusion of nutrients provided by the mother plant and support symplastic transport [11,56,57]. The high metabolic activity of parenchymatic cells is supported by detected peroxidases activity (Figure 6C). When seed development is completed, the major role of the SC is to protect the embryo. This is achieved via structural, chemical composition, and enzymatic activities [1,16]. In legumes, the SC has an additional role in regulating the germination timing via SC permeability and this trait has been targeted during the domestication process [13,15]. The physical dormancy, particularly widespread among legume species, is mediated by SC properties [13,37,41,51,58]. However, the process of dormancy establishment and release in legume seeds is still not well understood [13]. The impregnation of SC by phenolic and lipid substances is hypothesized to play a role [30,31,41]. The oxidation and polymerization of phenolic substances is regulated by oxidation via peroxidases and/or polyphenol oxidases [30,59,60,61,62]. There is ongoing debate over the causal substances, as in some species, these have been attributed to the deposition of callose or suberin in the macrosclereids or phenolic compounds, while others did not support this [30,31,37,54,63]. Although Spurný has not conducted a thorough developmental analysis [25,26,27,28], he sampled seeds at various stages of maturity and observed the gradual decrease in SC permeability to water even in cultivated (e.g., non-dormant) pea genotype. He followed in more detail the development of macrosclereids, which were in agreement with an earlier report [24]. The macrosclereids are derived from protoderm by anticlinal division and gradually elongate, and their secondary wall undergoes characteristic thickening. Spurný (1963) claims that there are suberin deposits on the macrosclereids surface [27], however, our previous analyses did not confirm that [37,41,51]. On the other hand, the results from SR staining point to the presence of lipid substances, supported by the detection of differentially abundant hydroxylated fatty acids present in dormant [41,49,51], wild peas such as JI64 and JI1794. Although our current study was not primarily testing SC permeability and resulting seed dormancy, the comparisons between wild and cultivated pea genotypes show the differences in SC structure and histochemistry. The presence of polyphenolics (proanthocyanidins, PAs) during the pea SC development was documented earlier using histochemical staining and were confirmed by chemistry analysis [37,41,51]. In the present study, we indirectly analyzed PAs, as TB stain can be used as inference [43]. Similar to in *M. truncatula* SC, there is an increase in polyphenolic compounds during development and their deposition in vacuoles [19].

The SC has several regions, which are anatomically distinct, i.e., the hilum, the micropyle, and the raphe. The hilum is a scar formed by the detached funiculus at seed maturity. The micropyle is formed at the margins of the integuments and it forms the pore through which the radicle emerges during seed germination. The raphe is a slightly depressed area on the opposite side of the hilum from the micropyle. There is ongoing discussion about the processes and structures controlling water entry into legume seeds [13]. It was thought that the hilum or the micropyle is important for controlling water entry. Similarly, the lens was proposed to control the release of seed dormancy in some legumes but several species, including soybean and pea, do not have this feature. While the hilum and tracheid bar play a role in seed desiccation, it is not a major water entry point in peas during the imbibition [37]. In contrast to peas, in some legume species [64,65,66], the function of the hilum was experimentally confirmed to operate as a hygroscopically activated valve.

Peroxidase (PRX) activity was found to be related to lignification and the content of phenolics in the water-impermeable SC of legumes and Malvaceae species [59,60]. We detected PRX activity in pea SC, similar to findings in soybean [61,67], with increasing activity during development peaking at 21 DAA. PRX activity localized predominantly in the hilum region (Figure 6C,E). We detected high PRX activity in the cultivated pea, cv. Cameor, which does not have pigmented or impregnated SC. It has been suggested that soybean SC peroxidase is involved in extensin polymerization, lignification, or suberization [59,67], and recent work on *Arabidopsis* supports the role of PRX in lignin formation [68,69]. Interestingly, the production of reactive oxygen species (ROS) mediates germination by direct scissoring of cell wall polysaccharides [70]. Notably, no lignin was detected in the pea SC [31,37,45], although the sensitivity and specificity of histochemical staining might be limited. On the other hand, catechol/polyphenol oxidase (PPO) activity was detected during wild pea (*P. elatius*) seed maturation and was remarkably absent in cultivated (*P. sativum*) pea [30,31]. It is speculated that, similarly to *Arabidopsis* [71], the oxidation and polymerization of phenolic compounds might be responsible for SC impermeability [29]. Physical dormancy can be removed by mechanical and chemical scarification, but little is known about the natural process [41,72]. The role of temperature and soil humidity oscillations is hypothesized and has been tested and confirmed in wild pea [72]. The action of other factors, including soil microbes, passage through the digestive tract, soil chemistry, and abrasion cannot be excluded.

## 4. Conclusions

The current study shows the major anatomical and histochemical differences during pea SC development. Anatomical differences between wild and cultivated pea genotypes, particularly the length of macrosclereid cells, are clearly manifested already at the early stages of development. The wild pea SC was about twice as thick as the SC of cultivated genotypes. Young stages (13–20 DAA) are marked by strong metachromatic staining, indicative of high metabolic activity and the synthesis of secondary metabolites, particularly in wild pea SC. SR staining of lipidic substance was detected above the LL in the upper part of macrosclereid cells in all development stages of wild pea genotypes. Polyanionic substances stained by RR were localized to the upper part of macrosclereid cells and to the lamella between macrosclereid cells. Upon seed imbibition, RR staining was detectable across the SC in cultivated peas, while in wild genotypes only in macrosclereid cups. High peroxidase activity was detected in both wild and cultivated genotypes and increased over development with a peak prior to desiccation. A detailed knowledge of anatomy is important for any molecular or biochemical studies, including gene expression and proteomic analysis, especially when comparing different genotypes and treatments. Analysis is useful for other pairs of crop–wild progenitors of phylogenetically related and economically important legume crops, such as chickpea and lentil.

## 5. Materials and Methods

### 5.1. Plant Material

Cultivated pea cv. Cameor (reference for pea genome sequence), primitive domesticated landrace JI92 (Afghan type, non-dormant), and wild pea (*P. sativum* subsp. *elatius*) JI64, JI1794 (Turkey, dormant) were used [37,41]. Seeds were harvested from plants grown in 5 L pots with peat-sand (90:10) substrate mix (Florcom Profi, BB Com Ltd., Letohrad, Czech Republic) and fertilized weekly (Kristalon Plod a Květ, Agro Ltd., Říkov, Czech Republic. Plants were grown in glasshouse conditions (January–May 2019, 2020) with a day/night temperature of 35–20/18–12 °C, and supplemented with light (Sylvania Grolux 600 W, Hortilux Schreder, Holland) to extend the photoperiod to 14 h. Mature seeds were manually harvested, air dried, and stored at 24–22 °C in a dark and dry place until analysis. The dormancy was measured by a standard method [41].

### 5.2. Developmental Stages Labeling

Randomly selected flowers were tagged on the day of opening (considered day zero). Subsequently, the material was followed and harvested at selected time points, labeled as days after anthesis (DAA; from 10 to 30). Seeds were dissected, fixed in 1% formaldehyde in phosphate buffer (0.1 M pH 7.2), and stored at 4 °C. Ten DAA corresponds to an early stage (cell division and morphogenesis); 13, 21, and 27 DAA to middle stages (cell expansion and seed filling); and 30 DAA to physiological maturity. In 13 DAA seeds, the torpedo embryo occupies a relatively small fraction of the seed volume, the remainder of the embryo sac cavity being filled with liquid endosperm; the dry weight of seeds is 18–23% of the fresh weight. By 21 DAA, the surface of the mature embryo is in contact with the seed coat and storage compounds accumulation is ongoing; the dry weight of seeds is 26–32% of the fresh weight. By the end of seed development at 30 DAA, the accumulation of storage compounds (proteins and saccharides, largely starch) is finished and the seed is desiccating; the dry weight of the seeds is 48–56% of the fresh weight.

### 5.3. Anatomical and Histochemical Analysis

Samples of seed coat were dissected from dry (mature seeds) or fixed seeds, and saturated with 2% sucrose solution under vacuum for 1 h. Thereafter, equal volume of cryo-gel (Cryo-gel Leica, REF: 39475237) was added to samples. Saturated samples were mounted into cryo-gel on the alum chuck, frozen down to −20 °C, and cut in cryotome (Leica CM1950, Leica Microsystems Europe, Breckland, UK) into 15–20 µm transversal sections [43]. Notably, our analysis was conducted on cry-sectioned material without any dehydration of wax/plastic embedding steps, which might modify SC properties. Sections were stained with Toluidine Blue O (0.01%, *w*/*v* in water; Sigma-Aldrich Ltd., Prague, Czech Republic), Sudan Red 7B (0.01%, *w*/*v*; Sigma Aldrich, CZ), or Ruthenium Red (0.01%, *w*/*v* in water; Sigma-Aldrich Ltd., Prague, Czech Republic) according to Soukup [37,43] and observed with an Olympus BX 51 microscope (Olympus Corp., Tokyo, Japan) in bright field. Seed surface was observed under a 3D digital microscope VHX 7000 (Keyence International, Mechelen, Belgium). The histochemical detection of peroxidase activity was performed by chloronaphthol staining according to Gijzen et al. [61] with slight modifications. In brief, segments of freshly dissected seed coats were cut on cryomicrotom (50 µm) and immediately stained in chloronaphthol staining solution, which was prepared from 0.05% 4-chloro-1-naphthol (Sigma Aldrich, CZ) in 20 mM potassium phosphate buffer pH 7.0. First of all, 1.5 mg of 4-chloro-1-naphthol was diluted in 1 mL of ice-cold methanol, followed by adding 2.7 mL of 20 mM potassium phosphate buffer pH 7.0. Before use, 1 mL of chloronaphthol solution was mixed with 20 µl of 30% hydrogen peroxide. After 10 min staining in this solution, the segments were washed in potassium phosphate buffer for 2 min. Finally, the segments were observed by bright-field microscopy in 50% glycerol. Figures were documented with an Apogee U4000 digital camera (Apogee Imaging Systems, Inc., Roseville, CA, USA). Quantitative evaluation of selected anatomical traits (cell wall thickness and cell dimensions) was carried out on five independently sectioned seeds using ImageJ processing program [73]. Five sections per seed were measured in triplicate.

## Data Availability

Not applicable.
